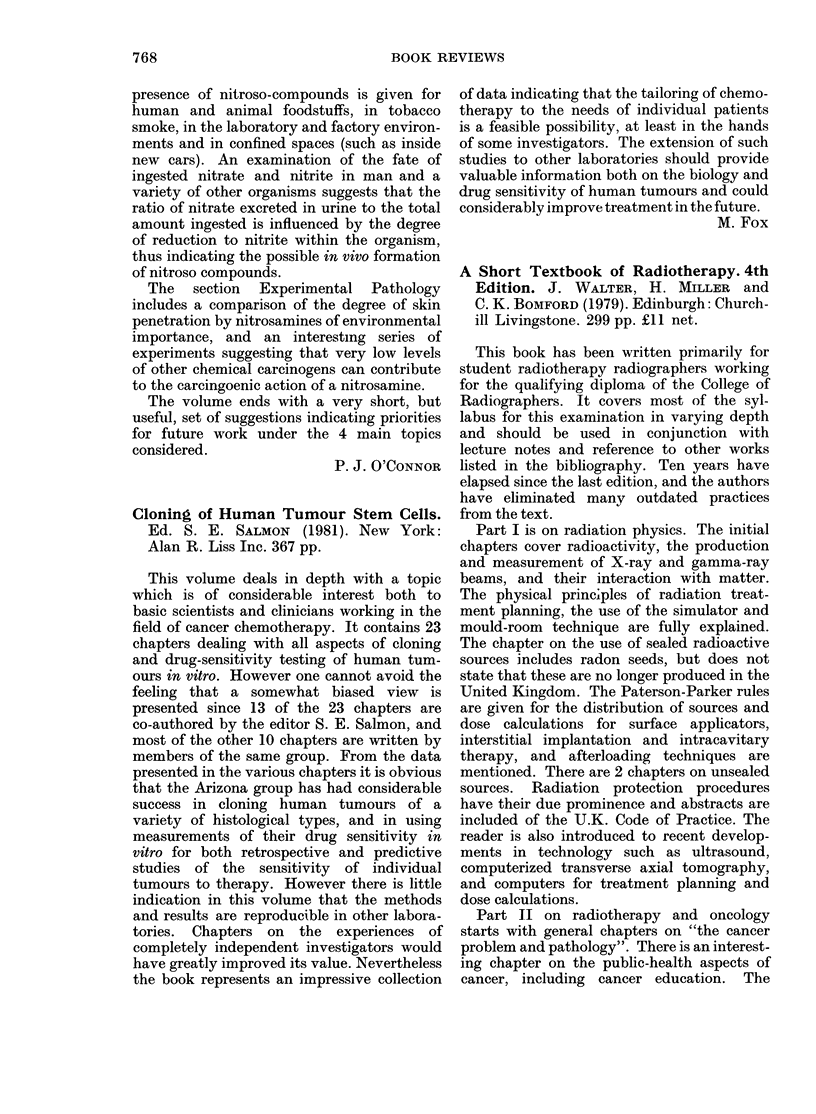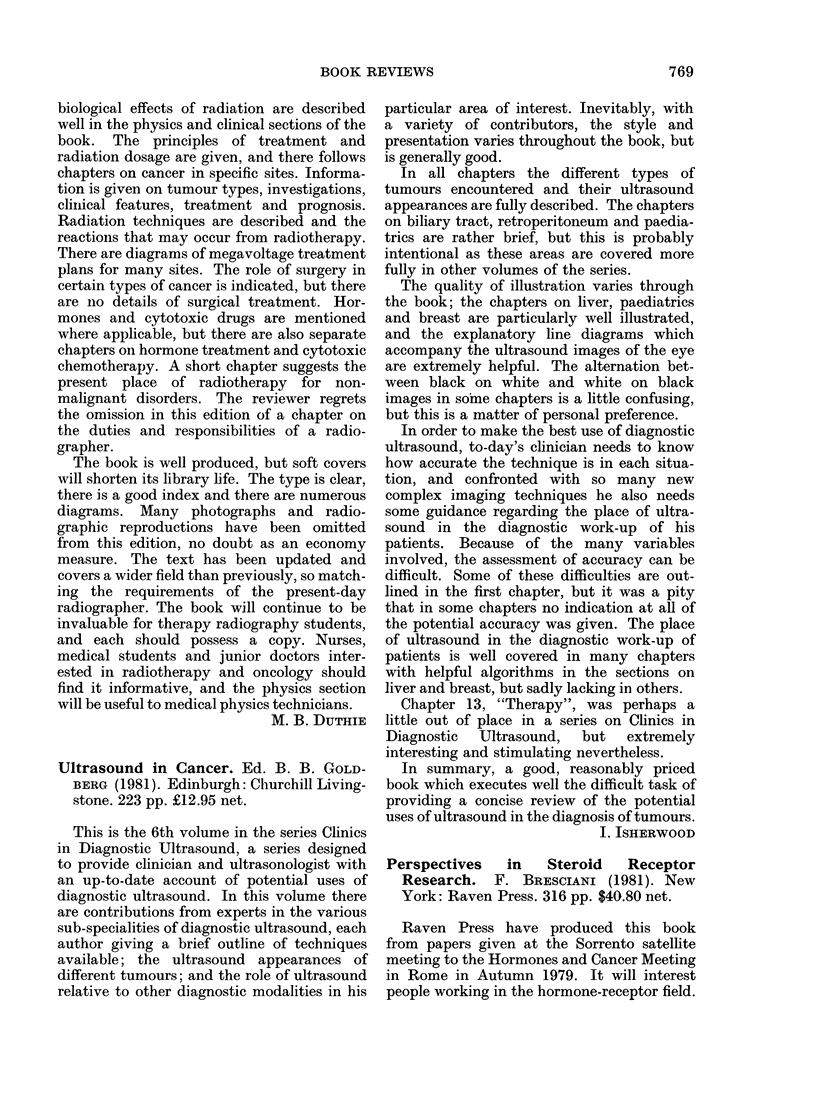# A Short Textbook of Radiotherapy. 4th Edition

**Published:** 1981-11

**Authors:** M. B. Duthie


**A Short Textbook of Radiotherapy. 4th Edition.** J. WALTER, H. MILLER and C. K. BOMFORD (1979). Edinburgh: Churchill Livingstone. 299 pp. £11 net.

This book has been written primarily for student radiotherapy radiographers working for the qualifying diploma of the College of Radiographers. It covers most of the syllabus for this examination in varying depth and should be used in conjunction with lecture notes and reference to other works listed in the bibliography. Ten years have elapsed since the last edition, and the authors have eliminated many outdated practices from the text.

Part I is on radiation physics. The initial chapters cover radioactivity, the production and measurement of X-ray and gamma-ray beams, and their interaction with matter. The physical principles of radiation treatment planning, the use of the simulator and mould-room technique are fully explained. The chapter on the use of sealed radioactive sources includes radon seeds, but does not state that these are no longer produced in the United Kingdom. The Paterson-Parker rules are given for the distribution of sources and dose calculations for surface applicators, interstitial implantation and intracavitary therapy, and afterloading techniques are mentioned. There are 2 chapters on unsealed sources. Radiation protection procedures have their due prominence and abstracts are included of the U.K. Code of Practice. The reader is also introduced to recent developments in technology such as ultrasound, computerized transverse axial tomography, and computers for treatment planning and dose calculations.

Part II on radiotherapy and oncology starts with general chapters on "the cancer problem and pathology". There is an interesting chapter on the public-health aspects of cancer, including cancer education. The biological effects of radiation are described well in the physics and clinical sections of the book. The principles of treatment and radiation dosage are given, and there follows chapters on cancer in specific sites. Information is given on tumour types, investigations, clinical features, treatment and prognosis. Radiation techniques are described and the reactions that may occur from radiotherapy. There are diagrams of megavoltage treatment plans for many sites. The role of surgery in certain types of cancer is indicated, but there are no details of surgical treatment. Hormones and cytotoxic drugs are mentioned where applicable, but there are also separate chapters on hormone treatment and cytotoxic chemotherapy. A short chapter suggests the present place of radiotherapy for non-malignant disorders. The reviewer regrets the omission in this edition of a chapter on the duties and responsibilities of a radiographer.

The book is well produced, but soft covers will shorten its library life. The type is clear, there is a good index and there are numerous diagrams. Many photographs and radiographic reproductions have been omitted from this edition, no doubt as an economy measure. The text has been updated and covers a wider field than previously, so matching the requirements of the present-day radiographer. The book will continue to be invaluable for therapy radiography students, and each should possess a copy. Nurses, medical students and junior doctors interested in radiotherapy and oncology should find it informative, and the physics section will be useful to medical physics technicians.

M. B. DUTHIE